# Effects of Levosimendan on cardiac function, size and strain in heart failure patients

**DOI:** 10.1007/s10554-020-02077-z

**Published:** 2020-10-24

**Authors:** D. Beitzke, F. Gremmel, D. Senn, R. Laggner, A. Kammerlander, A. Wielandner, R. Nolz, M. Hülsmann, C. Loewe

**Affiliations:** 1 Department of Biomedical Imaging and Image-guided Therapy, Medical University of Vienna, Waehringer Guertel 18-20, 1090 Vienna, Austria; 2 Department of Surgery, Medical University of Vienna, Vienna, Austria; 3 Department of Orthopedics and Trauma Surgery, Medical University of Vienna, Vienna, Austria; 4 Department of Internal Medicine II / Division of Cardiology, Medical University of Vienna, Vienna, Austria

**Keywords:** Heart failure, Cardiac magnetic resonance, Strain imaging, Levosimendan

## Abstract

Levosimendan improves cardiac function in heart failure populations; however, its exact mechanism is not well defined. We analysed the short-term impact of levosimendan in heart failure patients with ischemic and non-ischemic cardiomyopathy (CMP) using multiparametric cardiac magnetic resonance (CMR). We identified 33 patients with ischemic or non-ischemic CMP who received two consecutive CMR scans prior to and within one week after levosimendan administration. Changes in LV ejection fraction (LVEF) and LV volumes, as well as changes in strain rates, were measured prior to and within one week after levosimendan infusion. LV scarring, based on late gadolinium enhancement (LGE), was correlated to changes in LV size and strain rates. Both LV endiastolic (EDV) and endsystolic volumes (ESV) significantly decreased (EDV: p=0,001; ESV: p=0,002) after levosimendan administration, with no significant impact on LVEF (p=0.41), cardiac output (p=0.61), and strain rates. Subgroup analyses of ischemic or non-ischemic CMP showed no significant differences between the groups in terms of short-term LV reverse remodeling. The presence and extent of scarring in LGE did not correlate with changes in LV size and strain rates. CMR is able to monitor cardiac effects of levosimendan infusion. Short-term follow-up of a single levosimendan infusion using CMR shows a significant decrease in LV size, but no impact on LVEF or strain measurements. There was no difference between patients with ischemic or non-ischemic CMP. Quantification of LV scarring in CMR is not able to predict changes in LV size and strain rates in response to levosimendan.

## Introduction

The inodilator levosimendan is a calcium sensitizer used in patients with acute, decompensated heart failure or in the setting of cardiac surgery for ischemic heart disease or valvular heart disease [1]. In addition to the positive inotropic effects, anti-inflammatory, anti-oxidant, and cardioprotective effects are presumed that directly affect the heart [2]. On top of these cardiac effects, the peripheral resistance is lowered as a consequence of levosimendan-induced vasodilation [2]. Which system—the heart or the peripheral system—is predominantly affected is still a matter of debate, and therefore, the use of levosimendan in acute heart failure patients remains controversial. Some prospective studies favor drug usage, whereas other randomized trials do not support the advantages of levosimendan therapy [1, 3–5]. An overall definitive advantage of levosimendan administration is lacking, but there might be subgroups of patients who would benefit from its use [6]. Predictors of hemodynamic response to drug exposure also remain unclear [7].

Cardiac magnetic resonance (CMR) is a well-established tool for the assessment of cardiac function und structure. CMR is considered the gold standard in the assessment of ventricular function and allows for accurate scar quantification based on late gadolinium enhancement (LGE) [8]. The presence or absence of scarring in non-ischemic dilated cardiomyopathy (CMP), as well as quantification of infarct size and transmurality in ischemic cardiomyopathy (ICMP) , is already an independent risk factor for treatment success in case of revascularization [9, 10]. The presence of LGE is also associated with the decreased ability for reverse remodeling in LV dilatation [11].

Recent advances in CMR feature tracking techniques also allow for the evaluation of myocardial deformation. The technique is based on identifying the motion of cardiac structures through the cardiac cycle. From the assessment of these movements, longitudinal and circumferential strain values can be derived that provide information about myocardial fiber function [12]. Here, global longitudinal strain (GLS) measurements are more representative of subendocardial fiber function, whereas global circumferential strain (GCS) measurements are more representative of subepicardial fiber function [12]. The technique has already undergone validation in terms of reproducibility, and reduced GLS (derived by echocardiography and CMR) has also been proven to be an independent risk factor in patients with dilated cardiomyopathy (DCM) [13, 14]. Furthermore, strain measurements could allow for monitoring of drug interventions or revascularization procedures [15].

Therefore, the aim of this analysis was to evaluate the impact of levosimendan on cardiac function, structure, and deformation, assessed CMR in a heart failure population.

## Materials and methods

All patients who received levosimendan prior to planned heart surgery for valvular and/or coronary artery bypass grafting between March 2010 and February 2018 were identified via the institutional pharmacy records. This cohort was scanned for the presence of two consecutive CMR scans that were obtained during the clinical routine work-up for the evaluation of LV function, size, viability assessment and presence of scarring.

Overall, 54 patients were identified who received levosimendan in the analysis period. Seven patients were excluded due to the lack of two consecutive CMR (three patients with a pacemaker, four patients with other contraindications for CMR). Another five patients were excluded due to right heart failure based on pulmonary or tricuspid valve insufficiency.

## CMR

All patients were scanned on a 1.5T system (Siemens Avanto Fit; Siemens Healthineers; Erlangen; Germany). The CMR imaging protocol included steady state free precession (SSFP) imaging in two-, three-, and four-chamber views, using the left ventricular outflow tract and short axis for the evaluation of cardiac function (TR 3.2ms, TE 1.2ms; flip angle, 64°; voxel size, 1.4x1.4x36 mm; matrix 180x256 pixels, slice thickness 8mm). Phase contrast angiography was performed in patients with aortic insufficiency in slices perpendicular to the ascending aorta at the sinotubular junction. Typical parameters were: TR: 4.8 ms; TE: 2.8 ms; matrix: 320 × 300 mm; flip angle: 12°; temporal resolution: 25 to 55 ms; and velocity window: 2.5 to 4.0 m/s depending on the presence or absence of alaising. LGE was performed in the short axis, with two-, three-, and four-chamber views, 10 to 15 minutes after the injection of 0.15 ml gadobutrol (Gadovist®, Bayer-Schering, Austria) per kg bodyweight using T1-weighted, phase-sensitive, inversion-recovery (PSIR) sequences (TR: 849.6 ms; TE:1.07ms; voxel size 1.4x1.4x3.8 mm; 146x256 matrix, slice thickness 8mm; distance factor 0%). A second CMR scan without LGE was conducted after the administration of levosimendan.

## CMR postprocessing

For CMR evaluation, LV volumes and function were derived from short axis Cine SSFP, whereas scarring was determined from the LGE images. To quantify both functional and scar parameters, a commercially available post-processing software was used (QMass, Medis; Leiden, NL). LV function was derived from short axis Cine SSFP images in diastole and systole according to valid recommendations [16]. Positive LGE was analyzed using the full width at half maximum method [17]. Regurgitant fraction in patients suffering from aortic insufficiency was analysed by placing a region of interest at the vessel borders of through plane phase contrast angiography [16].

Forty-two patients were included in the advanced CMR postprocessing analysis (QStrain, Medis; Leiden, NL). For the assessment of global longitudinal strain (GLS) and global circumferential strain (GCS), long axis and short axis Cine SSFP were analyzed using a recently developed feature-tracking software (QStrain, Medis, Leiden, NL). During the analysis, another nine patients had to be excluded due to poor image quality/motion artifacts (n=4), lack of LGE (n=1), or incompatibility of the CMR scans with the feature-tracking postprocessing software (n=4).

## Patient population

Ultimately, 33 patients were included for analysis. Patient characteristics are listed in Table 1. All patients received standard heart failure therapy (Table 1).

**Table 1:** Clinical characteristics of patients receiving levosimendan

Demographics	
Male Sex (n)	30 (91%)
Age (years)	62a (50–79a)
Risk factors	n (%)
Hypertension	30 (91)
Diabetes mellitus	13 (39)
Hyperlipidemia	19 (58)
(History of) Smoking	15 (45)
BMI >25	
Etiology of heart failure	n (%)
Ischemic cardiomyopathy	17 (52)
Valvular disease	10 (30)
Ischemic cardiomyopathy and valvular disease	6 (18)
Medication	
Beta blocker	30 (91)
ACE/ATII Inhibitors	32 (97)
Spironolactone	29 (88)
Diuretics	16 (48)
Digoxin	1 (3)

ICMP, defined by the presence of subendocardial LGE, was present in 17 patients (52%), and valvular disease was observed in 10 patients (30%). In the latter group, three patients presented with severe aortic stenosis, four patients presented with aortic insufficiency, two patients had an isolated mitral valve insufficiency, and one patient presented with a combination of an aortic stenosis and a severe mitral valve insufficiency. A combination of ICMP and valvular disease was observed in six patients (18%; aortic stenosis n=2; aortic insufficiency n=2; mitral valve insufficiency n=2). In patients with aortic insufficiency mean phase contrast derived regurgitant fraction was 42.5% (SD 17.8%; range 14–72%).

## Levosimendan administration

Levosimendan was administered via a 24h lasting infusion (0.1 mcg/kg/min) without the application of a starting bolus under hemodynamic monitoring in an intermittent care setting. Brain natriuretic peptide levels were assessed before and 3 days after the infusion.

## CMR post levosimendan

Three to seven (mean 3.8) days after levosimendan infusion, a second CMR without LGE was performed to evaluate changes in LV size, function, and strain. All analyses were performed by experienced CMR readers (RL, DS), in consensus, strictly according to the recommendations of the society of cardiac magnetic resonance [16]. Consecutive scans from each patients were analysed within a single session.

## Statistical analysis

All statistical analyses were performed using SPSS for Windows (version 23.0; IBM Corporation, Somers, NY, USA). Baseline characteristics are displayed as mean±SD, or median (IQR), and total numbers (%), as appropriate. Normal distributed parameters of LV function, size, and strain parameters were compared using a paired T-test. Non-normally distributed parameters (NT-proBNP) were compared using the paired Wilcoxon test.

We stratified patients according to the presence or absence of post-ischemic scarring on LGE, defined by the presence of a subendocardial scar and a scar volume of more than 5% of the LV mass. In this subgroup analysis, not only the absolute values of the LV parameters, but also the delta values of the parameters were compared between the groups using a univariate ANOVA. A subgroup comparison of NT-proBNP was performed using the Kruskal-Wallis test. Furthermore, the potential impact of scar volume on changes in LV function was assessed by a correlating the LV scar volume (%) to the Δ of LV functional parameters using a Pearson correlation.

## Results

### Impact of levosimendan on LV function

**Fig. 1:**
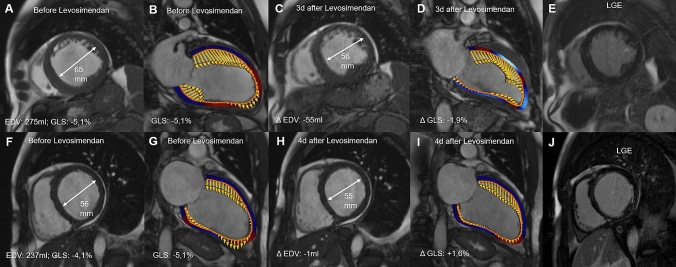
CMR before and after Levosimendan infusion.**b**–**e** shows a decrease in LV size after Levosimendan accompanied by an improvement in strain rate in a patient with aortic insufficiency and no evidence for LV scarring.

Mean end diastolic volume (EDV), and mean end systolic volumes (ESV) significantly decreased after levosimendan infusion (mean EDV before levosimendan 259.8 ± 87.6 ml to 245.1 ± 87.7 ml [103.63–437.7] after the infusion –p = 0.001; mean ESV before levosimendan 187.1 ± 68 decreased to 174 ± 71.6 ml [54.6–343.9] – p = 0.002) (Fig. 1). All other CMR-derived LV functional parameters did not change significantly (stroke volume [SV] from 72.7 ± 33.5ml to 71.1 ± 33.1ml; ejection fraction from 28.3 ± 8.8% to 29.2 ± 9.4% before and after infusion, respectively). Cardiac output (CO) (5 ± 2.3l/m^2^ to 5.1 ± 2.3l/m^2^), global longitundinal strain (GLS) (−7.8 ± 4% to − 8.3 ± 4%), global circumferential strain (GCS) (−12.3 ± 5.5% to −12.4 ± 5.6%), and global radial strain (GRS) (16.7 ± 8.9 to 17.6 ± 8.8) also remained unaffected. Results are shown in Table 2.

**Table 2:** Results for left ventricular volumetry, ejection fraction, strain parameters, and brain natriuretic peptide levels prior to and after levosimendan infusion

LV Parameter	Pre levosimendan	Post levosimendan	p
EDV (ml)	259.8 ± 87.6 [113.3–457.6]	245.1 ± 87.7 [103.63–437.7]	**0.001**
ESV (ml)	187.1 ± 68 [72.7–339.1]	174 ± 71.6 [54.6–343.9]	**0.002**
SV (ml)	72.7 ± 33.5 [28.6–168.6]	71.1 ± 33.1 [22.4–159.5]	0.59
EF (%)	28.3 ± 8.8 [12.9–48.6]	29.2 ± 9.4 [12.9–47.3]	0.41
CO (l/min)	5 ± 2.3 [2.1–11.6]	5.1 ± 2.3 [2.2–11.7]	0.61
GLS	− 7.8 ± 4 [−17.9 to − 0.82]	− 8.3 ± 4 [− 14.9 to − 1.4]	0.09
GCS	-12.3 ± 5.5 [− 27.1–0.27]	− 12.4 ± 5.6 [− 27 to − 2.7]	0.69
GRS	16.7 ± 8.9 [2.7–45.1]	17.6 ± 8,8 [4.1–38.6]	0.14
NT-proBNP ng/ml	5778.5 ± 7623.5 [432–35000]	4017.9 ± 5652.4 [121.9–25533]	**0.002**
LGE (% of LV)	16.9 ± 11.9 [0–45,9]		N/A

*LV* left ventricle, *EDV* endiastolic volume, *ESV* endsystolic volume, *SV* troke volume, *EF* ejection fraction, *CO* cardiac output, *GLS* global longitudinal strain, *GCS* global cirumferential strain, *GRS* global radial strain, *NT pro BNP* N-terminal pro brain natriuretic peptide, *LGE* late gadolinium enhancement

NT-proBNP significantly decreased from a of mean 5778.5 ± 7623.5 pg/ml to 4017.9 ± 5652.4 pg/ml (p = 0.002).

### Group comparison of patients with and without Ischemic scar burden

Twenty-three patients presented with subendocardial scars (ICMP), and/or a combination of ICMP and valvular disease. Ten patients showed LV dilatation based on valvular disease only, but without any post-ischemic, subendocardial scar. Results of a group comparison of these two patient cohorts is shown in Table 3. In patients with ICMP, GLS was significantly lower (6.8 ± 4 vs. − 9.9 ± 3.3; p = 0.04) prior to levosimendan administration. After levosimendan, this significance vanished. The Δ for GLS changes between the groups showed no significant difference. No other parameters showed any difference between the groups prior to or after levosimendan infusion.

**Table 3:** Subgroup analysis of left ventricular parameters and changes after levosimendan in patients with ischemic cardiomyopathy and valvular disease

LV Parameter	ICMP			Valve Disease			p between ICMP and valve disease prior to levosimendan	p between ICMP and valve disease post levosimendan	p Delta
	Before levosimendan	Post levosimendan	Delta	Before levosimendan	Post levosimendan	Delta			
EDV	256.3 ± 89.8 [113.3 – 457.6]	239.7 ± 87.9 [103.6 – 437.7]	17.9 ± 23.6 [−7.3 − 83.9]	267.7 ± 86.3 [122.2 – 382.5]	257.6 ± 90.8 [108.3 – 369.28]	10.1 ± 14.2 [−13.2–25.8]	0.74	0.6	0.34
ESV	190 ± 72.2 [72.7 – 339.1]	174 ± 78.3 [54.6 ± 343.9]	17. ± 23.9 [−27.3 – 52.4]	180.4 ± 59.8 [88.1–238]	174.2 ± 56.6 [83.7 – 234.3]	7.6 ± 10.6 [−10.65–28.3]	0.71	0.99	0.24
SV	66.3 ± 30.4 [28.6 – 168.6]	65.8 ± 24.8 [37 – 138.8]	− 0.9 ± 8.1 [−18.3 − 22.7]	87.3 ± 37.1 [34 –144.5]	83.4 ± 46.3 [22.4–159.5]	4 ± 16.4 [−23.7–25.5]	0.1	0.16	0.9
EF	26.5 ± 8.2 [12.9 – 47.4]	28.4 ± 9 [12.9 – 7.3]	3.9 ± 19.6 [−27.3 – 52.4]	32,6 ± 9 [17.2 – 48.6]	31.1 ± 10.4 [13.6 – 43.2]	1.5 ± 5.5 [−5.9 –10.5]	0.06	0.46	0.41
CO	4.5 ± 2.1 [2.1 – 11.6]	4.7 ± 1.8 [2.7 – 9.6]	0.1 ± 1.6 [−2.51 – 5.5]	6.1 ± 2.5 [2.5 − 10.5]	6 ± 3.2 [2.2 – 11.7]	0.08 ± 1.1 [−1.73 – 2.12]	0.071	0.14	0.9
GLS	− 6.8 ± 4 [−17.9 − − .82]	− 7.7 ± 3.9 [−14.9 − 1.38]	0.9 ± 1.7 [−3.7 − 3.13]	−9.9 ± 3.3 [−14.8 – −6.2]	− 9,7 ± 3,8 [−14,86 − 4,3]	0.2 ±1.6 [3.5 –1.7]	**0.04**	0.2	0.1
GCS	− 1.2 ± 5.5 [−27.14 − − .27]	− 11.6 ± 5.9 [−27 − − 2.7]	0.4 ± 2.6 [−5,3 − 4,1]	−14.7 ± 4.9 [−21.5– −6.3]	− 14.3 ± 5 [−22.5 – −6.34]	0.4 ± 2 [−4.95–2]	0.1	0.21	0.4
GRS	14.8 ± 8.9 [2.7 – 45.1]	16.2 ± 8.8 [4.1 − 38.6]	− 1.4 ± 3 [−5,7 – 6.5]	21.2 ± 7.7 [11,. – 31.4]	20.6 ± 8.5 [8.2 – 34.5]	0.5 ± 2.4 [−3.1 – 3.9]	0.06	0.19	0.1
NT-proBNP	5778.5 ± 7623.5 [432.1 – 35000]	3432.6 ± 4630.5 [121.9 – 22615]	1794.2 ± 3191.4 [− 2268 − 12385]	7047.4 ± 8340.6 [525.3 – 27418]	5364 ± 7635.4 [248–25533]	1683.3 ± 2442.1 [−2817 − 6370]	0.7	0.94	0.92

*ICMP* ischemic cardiomyopathy, *LV* left ventricle, *EDV* endiastolic volume, *ESV* endsystolic volume, *SV* stroke volume, *EF* ejection fraction, *CO* cardiac output, *GLS* global longitudinal strain, *GCS* global cirumferential strain, *GRS* global radial strain, *NT* – pro BNP N-terminal pro brain natriuretic peptide

### Correlation between LV scarring and changes in LV function

Table 4 shows the results from the Pearson correlation between the percentage of LV scarring and changes in LV size, function, and strain. Overall, the percentage of LV scar in the study population showed no correlation to the changes in the functional parameters nor to the changes in LV size.

**Table 4:** Correlation between changes in left ventricular parameters with cardiac magnetic resonance and the percentage of left ventricular scar load by late gadolinium enhancement

	Δ	Pearson r	p
Scar %	ΔEDV	0.21	0.25
	ΔESV	0..24	0.17
	ΔSV	0.13	0.45
	ΔEF	0..08	0.67
	ΔCO	0.07	0.71
	ΔGLS	− 0.004	0.98
	ΔGCS	− 0.06	0.73
	ΔGRS	0.03	0.89
	ΔNT-pro BNP	− 0.16	0.374

*EDV* endiastolic volume, *ESV* endsystolic volume, *SV* stroke volume, *EF* ejection fraction, *CO* cardiac output, *GLS* global longitudinal strain, *GCS* global cirumferential strain, GRS global radial strain, *NT–pro BNP* N-terminal pro brain natriuretic peptide

### Discussion

In this retrospective analysis using multiparametric CMR, we were able to monitor the response to levosimendan infusion in patients with systolic heart failure. In the study cohort, a single dose of levosimendan resulted in significant reduction in LV size, shown by a significant decrease in LVEDV and LVESV (Fig 1). This resulted in unchanged LVEF values, as well as a non- significant increase in CO.. Most importantly, in this cohort, we could not show a significant increase in deformity parameters that might be representative of the positive inotropic effects of the drug on the LV myocardium using CMR strain imaging. Despite the fact that baseline GLS was significantly reduced in patients with ICMP when compared to patients with valvular disease, levosimendan infusion had no effect on LV strain values. Furthermore, the absolute amount of LV scarring assessed by LGE showed no correlation to changes in LV size, function, and strain after levosimendan infusion. Therefore, the amount of LV scaring seems to have no clinical impact on short-term treatment response to levosimendan infusion.

Levosimendan for the treatment of acute heart failure has been shown to be safe and efficient compared to placebo and dobutamine [1, 18]. However, its usefulness in the pre- or post-operative setting in in patients with low EF and/or conditions of acute heart failure in clinical practice remains controversial, as randomized studies have shown no impact on the length of intensive care unit stay or mortality [3, 4]. Therefore, appropriate indications and patient selection, as well as timing of the infusion in the setting of potential cardiac surgery, might be of clinical importance [6]. From available imaging modalities to date, only echocardiography has been used to monitor LV function with the use of the drug [19, 20]. With the exception of a case report, CMR, including tissue characterization for treatment monitoring, has not been reported in the literature thus [21].

In our cohort, using CMR, treatment response to levosimendan resulted in a significant decrease in LV size after a single infusion over 24 hours. This resulted in only a non-significant change in LV EF. These findings with CMR in our cohort differ from data reported from echocardiograms or studies using pulmonary catheterization [1, 22, 23]. This might be based on the difference in the evaluation of the EF primarily when using non-enhanced 2D echocardiography [24]. For example, Navarri et al. showed significant changes after levosimendan infusion in LVEF with echocardiography, but, at the same time, nonsignificant changes in LV systolic and LV diastolic values [22]. These finding are in contrast to ours and may be based on the methodology used for assessing LV volumes and LV EF. Imaging reports showed significantly lower EF values in echocardiography when comparing unenhanced echocardiography to CMR, and, despite a good correlation between the modalities, a wide limit of agreement in heart failure patients, especially with regard to LV volumes [25, 26]. This might favor the use of CMR in heart failure cohorts with severely dilated LVs. Furthermore, we were not able to exactly compare our study cohort to others published in the literature in terms of LV volumes, as most of the studies used invasive pulmonary artery catheterization to report on SV or solely reported on outcomes.

CMR, including LGE for quantification of scar load, has not been applied to response assessment or response prediction after levosimendan administration. In the literature, mostly invasive monitoring using pulmonary artery catheterization or echocardiography has been reported [1, 20]. In this cohort, LGE was assessed for myocardial viability or for diagnostic proposes in the assessment of LV dilatation [9, 10]. We evaluated whether there was an influence of scar load on therapy response to levosimendan. The aim of this analyses was to screen for patients who might not profit from levosimendan because of high (ischemic or non-ischemic) scar burden, as this would have an impact on costs and might avoid potential complications of levosimendan infusion, such as hypotension and hypokalemia. However, in our cohort, we were not able to show a correlation between changes in LV parameters and the percentage of scar load. In addition, there was no difference between patients with known ischemic CMP and dilatative CMP based on the presence of valvular disease. These findings are similar to results published by Najjar et al., who showed positive hemodynamic effects of levosimendan in a heart failure cohort, but failed to identify predictors of the response to drug exposure [7]. Prospective studies are lacking about the influence of LV structure on treatment response or even outcomes.

The assessment of left ventricular strain parameters and changes in these parameters appear to be valuable predictors of treatment response in various cardiac diseases [12]. The methodology has already shown proven reproducibility with an acceptable inter- and intraobserver variability [14]. Therefore, strain measurements seem to be a valuable alternative to EF measurements, as these parameters might be more representative of the improvement in myocardial function and deformity than EF measurements based on the ratio between EDV and ESV alone [27]. Strain measurements, especially GLS, have already been proven as an independent predictor of outcome in CMP and non-ischemic DCM, showing that a reduction in strain of one standard deviation has a severe impact on the hazard ratio for cardiovascular events [28, 29]. In ischemic heart disease GLS and GCS changes in the early phase after myocardial infarction were predictive of outcome [15]. More data on changes in strain measurements after medical or vascular interventions are currently lacking in the literature. Despite significant changes in LV volumes after levosimendan infusion, no changes in CMR-derived strain parameters could be observed in our cohort. Therefore, the observed reduction in LV size in our cohort might be based solely on lowering the peripheral resistance and not based on changes in inotropy. Dalla et al. described in his model with septic patients receiving norepinephrine that changes in afterload and preload i.e. an increase in systemic and pulmonary vascular resistance, impact LV EDV, but not ejection fraction or longitudinal strain [30]. The lack of changes in strain parameters in this cohort could also be explained by a lack of ability in the improvement of deformity based on longstanding LV dilatation, presence of valvular disease, and the presence of fibrosis. The mean percentage of LV fibrosis was 16% in our study group and it has already been shown that the presence of LGE is associated with reduced strain parameters [14]. Furthermore, the presence of LGE had an impact on GLS values prior to levosimendan infusion in our ICMP subgroup. However, we could not prove a correlation between the absolute amount of scarring by LGE and the changes in GLS in this subgroup, and strain parameters numerically showed even larger changes in the ICMP group when compared to valvular disease. Therefore, LGE does not predict (but also does not prevent) treatment response to levosimendan infusion.

In DCM, imaging studies on short-term changes in LV size are lacking and results of long-term studies are somehow divergent. On the one hand, the presence of LGE has been shown to be a negative predictor of LV reverse remodeling in non-ischemic CMP [31]. On the other hand, Tayal et al. failed to identify the measurement of cardiac structure or cardiac strain as a marker for LV LV recovery in DCM [32]. In their work, contractile reserve, as evidenced by dobutamine stress echocardiography, was predictive for a reduction in LV size [32]. In our cohort, we observed significantly smaller LV volumes after levosimendan infusion regardless of the presence of LV scarring in both groups.

There are several limitations of our study. First, it is a retrospective, single-center cohort study with the focus on imaging parameters. Data on the outcome and/or impact of levosimendan infusion on intermediate and long-term outcome are not available. In addition, the sample size is small and patients were selected non-randomly by a single referring physician, which may have caused a patient selection bias for the analysis. On the other hand by this patient selection was more homogenous than in a multi center setting. Additionally data on hemodynamic changes in case of valvular disease (e.g. changes in regurgitant fraction) are missing as flow measurements have not been implemented in all CMR studies.

## Conclusion

Multiparametric CMR including strain imaging can be used to monitor effects of levosimendan infusion. In patients with ischemic or non-ischemic CMP, a significant reduction in LV size can be observed after a single dose of levosimendan. This was not accompanied by a significant change in LV deformity parameters evaluated by strain measurements. CMR imaging and quantification of myocardial scarring is not able to predict treatment response to a single dose of levosimendan.

F-J shows a patients with ischemic cardiomyopathy and a subendocardial scar in the lateral wall. CMR before and after levosimendan infusion shows no decrease in LV size and no changes in global longitudinal strai
